# Moderate and severe traumatic brain injury in general hospitals: a ten-year population-based retrospective cohort study in central Norway

**DOI:** 10.1186/s13049-022-01050-0

**Published:** 2022-12-09

**Authors:** Shavin Rahim, Eivor Alette Laugsand, Even Hovig Fyllingen, Vidar Rao, Rabea Iris Pantelatos, Tomm Brostrup Müller, Anne Vik, Toril Skandsen

**Affiliations:** 1 Department of Neuromedicine and Movement Science, Faculty of Medicine and Health Sciences, Norwegian University of Science and Technology (NTNU), 7491 Trondheim, Norway; 2 Department of Public Health and Nursing, Faculty of Medicine and Health Sciences, Norwegian University of Science and Technology (NTNU), 7491 Trondheim, Norway; 3 Department of Surgery, Levanger Hospital, Nord-Trøndelag Hospital Trust, 7600 Levanger, Norway; 4 Department of Surgery, St. Olavs Hospital, Trondheim University Hospital, 7006 Trondheim, Norway; 5 Department of Radiology and Nuclear Medicine, St. Olavs Hospital, Trondheim University Hospital, 7491 Trondheim, Norway; 6 Department of Circulation and Medical Imaging, Faculty of Medicine and Health Sciences, Norwegian University of Science and Technology (NTNU), 7006 Trondheim, Norway; 7 Department of Neurosurgery, St. Olavs Hospital, Trondheim University Hospital, 7006 Trondheim, Norway; 8 Clinic of Physical Medicine and Rehabilitation, St. Olavs Hospital, Trondheim University Hospital, Trondheim, Norway

**Keywords:** Traumatic brain injuries, Craniocerebral trauma, General hospitals, Trauma centers, Tertiary care centers, Referral and consultation, Mortality, Aged, 80 and over

## Abstract

**Background:**

Patients with moderate and severe traumatic brain injury (TBI) are admitted to general hospitals (GHs) without neurosurgical services, but few studies have addressed the management of these patients. This study aimed to describe these patients, the rate of and reasons for managing patients entirely at the GH, and differences between patients managed entirely at the GH (GH group) and patients transferred to the regional trauma centre (RTC group). We specifically examined the characteristics of elderly patients.

**Methods:**

Patients with moderate (Glasgow Coma Scale score 9–13) and severe (score ≤ 8) TBIs who were admitted to one of the seven GHs without neurosurgical services in central Norway between 01.10.2004 and 01.10.2014 were retrospectively identified. Demographic, injury-related and outcome data were collected from medical records. Head CT scans were reviewed.

**Results:**

Among 274 patients admitted to GHs, 137 (50%) were in the GH group. The transferral rate was 58% for severe TBI and 40% for moderate TBI. Compared to the RTC group, patients in the GH group were older (median age: 78 years vs. 54 years, *p* < 0.001), more often had a preinjury disability (50% vs. 39%, *p* = 0.037), and more often had moderate TBI (52% vs. 35%, *p* = 0.005). The six-month case fatality rate was low (8%) in the GH group when transferral was considered unnecessary due to a *low risk of further deterioration* and high (90%, median age: 87 years) when *neurosurgical intervention was considered nonbeneficial*. Only 16% of patients ≥ 80 years old were transferred to the RTC. For this age group, the in-hospital case fatality rate was 67% in the GH group and 36% in the RTC group and 84% and 73%, respectively, at 6 months.

**Conclusions:**

Half of the patients were managed entirely at a GH, and these were mainly patients considered to have a low risk of further deterioration, patients with moderate TBI, and elderly patients. Less than two of ten patients ≥ 80 years old were transferred, and survival was poor regardless of the transferral status.

**Supplementary Information:**

The online version contains supplementary material available at 10.1186/s13049-022-01050-0.

## Background

Traumatic brain injury (TBI) is frequent, with an estimated 4 000 hospital admissions per year in Norway [1]. In low-income countries, the incidence of TBI has increased due to more traffic accidents, while in high-income countries, more elderly people sustain TBIs due to falls [2]. Approximately 15% of all hospital-referred TBIs are moderate or severe [1], with outcomes varying from complete restitution to severe disability or death.

Several guidelines for the initial management of TBI in Scandinavia have been published in recent decades [3–5]. In 2008, the Scandinavian Neurotrauma Committee (SNC) published summarised guidelines for the prehospital management of severe TBI based on previously developed guidelines by the Brain Trauma Foundation (BTF) [4, 6]. These guidelines recommend that all cases of suspected severe TBI (defined by a Glasgow Coma Scale (GCS) score of 3–8) should be stabilised and transported promptly to the nearest neurosurgical department for specialised neurointensive management. Notably, the SNC and BTF have not addressed the management of elderly patients with TBI specifically, even though these patients present with a greater burden of comorbid conditions and a higher risk of death and may not benefit from aggressive neurosurgical treatment [7].

For patients with a TBI not considered severe, the decision on primary admission to the regional trauma centre (RTC) or the closest local trauma centre (general hospital (GH)) is based on evaluations performed by the prehospital emergency personnel, taking a range of factors into consideration. The benefit of direct transportation has been extensively studied, with conflicting results, and a systematic review did not find a clear benefit of direct transportation to an RTC in this patient group [8]. Hence, the Norwegian National Trauma Plan states that if transportation time to the RTC is 45 min or more, transportation to the closest trauma centre should be considered [9].

The initial in-hospital management of minimal, mild, and moderate TBI in adults is described in guidelines that were developed by the SNC in 2000 and revised in 2013 [3, 5]. According to the guidelines applied in the period of the current study, all patients with signs of moderate TBI should have an early head CT scan and be admitted to the hospital for further observation for ≥ 12 h. In cases with abnormal head CT scans, consultation with a neurosurgical department is recommended. However, the guidelines do not specify which patients should be managed at the GH and which patients should be managed in a neurosurgical department.

Most studies of treatment and outcome of moderate and severe TBI originate from university hospitals with neurosurgical services [10]; hence, there is a shortage of knowledge about patients managed entirely at GHs.

This study aimed to describe adult patients with moderate and severe TBI admitted to GHs in a defined health region, focusing on patients who were never transferred (the GH group). We investigated the rate of and reason for managing patients entirely at the GH and explored differences between patients managed entirely at the GH and patients transferred to the RTC for further treatment, including differences in survival. Furthermore, we aimed to specifically describe the transferral rate and survival in elderly patients.

## Methods

### Study setting and study participants

The study comprised patients admitted primarily to GHs without neurosurgical services in central Norway, one of the four health regions in the country. Central Norway covered a population of approximately 670,000 inhabitants in the study period, and the distance between the GHs and the RTC ranged from 42 to 348 km by car. At the time of the study, the region held two levels of hospitals [11]: seven GHs meeting the requirements for level III trauma centres (for details see Additional file 1: Table S1) and one level I/II trauma centre, St. Olavs Hospital, with a neurosurgical department, denoted the RTC. The RTC also serves as a GH for approximately 45% of the population in the region, and most patients admitted there with TBI in need of hospitalisation for more than 24 h are admitted to the neurosurgical department, regardless of TBI severity. Patients belonging to the catchment area of St. Olavs Hospital were therefore not included in this study. As the RTC and the GHs lie within the same health region in central Norway, the standards of the prehospital care were the same for all patients and were in line with the guidelines applicable at the time they were admitted [3–5].

### Study procedures and inclusion and exclusion criteria

Patients admitted to one of the seven GHs of the region between 01.10.2004 and 01.10.2014, with a main or secondary diagnosis registered as ICD-10 code(s) S06.1-S06.9 or S09.7-S09.9 (International Classification of Diseases, 10th revision), were screened for inclusion by review of their medical records. The ICD-10 coding system does not have unique codes for the severity of TBI, but moderate or severe TBI would most likely be coded as one or several of the abovementioned codes [12]. The *Trondheim moderate and severe (ms)TBI study* served as an additional source of patient identification. This is a prospective cohort study of all patients with moderate or severe TBI admitted to St. Olavs Hospital, including those transferred from GHs, from 01.10.2004 onwards [13].

We included patients with moderate and severe TBIs upon admission to the emergency department (ED), as well as patients with mild TBI in the ED who deteriorated to moderate or severe TBI during the acute phase to capture the entire burden of moderate and severe TBI managed at the GHs. TBI was classified as mild (GCS scores 14–15), moderate (GCS scores 9–13), or severe (GCS scores 3–8) [14]. Patients with a GCS score of 13 were included as moderate TBI because of the higher frequency of intracranial lesions and risk of adverse outcomes [15, 16], in accordance with the Head Injury Severity Scale, commonly used in Scandinavia [17]. The acute phase was defined as the time from admission to the GH to discharge from any ward responsible for the management of the trauma or related complications. If the patient was transferred to the RTC, discharge from the RTC denoted the end of the acute phase. A subsequent stay at a GH or rehabilitative institution was not considered part of the acute phase.

Intoxicated patients with an initially low GCS score who regained normal consciousness within 12 h were considered to have mild TBI and were excluded [18]. Patients who did not reside in Norway were excluded. Children < 17 years old were also excluded, as we have previously shown that most of these patients are transferred to the RTC [19]. Patients with a chronic subdural haematoma and patients where a diagnosis of TBI was considered unlikely or uncertain were also excluded (e.g., patients with stroke), as well as patients who presented to the ED > 72 h after the trauma.

Medical records and head CT scans for the entire acute phase were reviewed, and patients were categorised into patients managed entirely at the GH (GH group) and patients transferred to the RTC (RTC group).

### Study variables

Preinjury disability was classified as *yes* if the patient had any known concurrent condition that affected daily functioning at the time of injury and was further categorised into neurological condition (including dementia), alcohol abuse, cardiopulmonary disease, psychiatric disorder, substance abuse, cancer, developmental disorder, *other*, or *several*. Preinjury antithrombotic medication was recorded as *yes* if the patient was using platelet inhibitors or anticoagulants (including heparins or direct-acting oral anticoagulants).

Alcohol intoxication upon admission was recorded as *yes* if clinical suspicion was described in the medical records or if the patient had a measured serum ethanol ≥ 2.2 mmol/L [20]. Injury-related variables were the cause of injury (fall, motor vehicle accident, or *other cause* including violence), admission GCS score (the lowest recorded GCS score in the ED) and severity of TBI at admission (mild, moderate, or severe). In cases with prehospital intubation, the last recorded GCS before sedation was used. Trauma team activation (TTA) at admission, extracranial injuries, extracranial surgery, and admission to the intensive care unit (ICU) were also registered. For patients transferred to the RTC, any neurosurgical procedure performed, including intracranial pressure (ICP) monitoring, was recorded.

Head CT scans for the entire acute phase (first and later CT scans) were reviewed by a resident in radiology (EHF), who was blinded to the patients’ medical records except for the CT referral information. CT findings were recorded dichotomously as *yes* or *no* for the variables epidural haematoma (EDH), subdural haematoma (SDH), traumatic subarachnoid haemorrhage (tSAH), single contusion/haematoma, multiple contusions/haematomas, intraventricular haemorrhage, general oedema (one or both hemispheres, or cerebellum), skull fracture with or without impression, and intracranial air.

Whether the consultant neurosurgeon or resident in neurosurgery on call at the RTC was consulted while the patient was in the ED, or was consulted later, was registered. When the patient was not transferred to the RTC, the main reason for keeping the patient at the GH after stabilisation in the ED was derived from the patient’s medical records and assigned to one of four predefined categories: *unsalvageable* was used for patients with a low GCS score, bilateral fixed semidilated pupils and/or a head CT scan demonstrating signs of severe herniation. *Neurosurgical intervention considered nonbeneficial* was selected when the risk associated with a neurosurgical procedure or intensive care was considered to outweigh the potential benefits associated with these interventions. *Presumed low risk of further deterioration* was selected in cases where the patient had a relatively high/stable GCS score upon arrival and modest head CT findings or in cases where alcohol intoxication was suspected to influence the GCS score [18]. *Another reason* was selected when the reason for keeping the patient at the GH did not fall into the other specified categories, for instance, severe trauma to other organ systems requiring immediate surgery at the GH or cases suggestive of isolated traumatic axonal injury (TAI) assuming no need for ICP measurement.

Outcome variables were the length of the acute hospital stay, discharge destination (nursing home, own home, specialised rehabilitation, other rehabilitative institution, or another hospital), death during the acute phase (i.e., in-hospital fatality), and death within 6 months and 12 months postinjury (6- and 12-month fatality). The cause of in-hospital fatality was categorised as *intracranial hypertension* when the clinical and radiological characteristics indicated increased ICP resulting in tamponade. *Complications of TBI* was selected when the patient died from complications or severe neurological impairments secondary to TBI. *Other trauma,* or *unrelated to the injury,* was selected when death was caused by trauma to a different organ system or was deemed unrelated to the TBI (e.g., a myocardial ischaemic infarction).

### Statistical analyses

Descriptive data are reported as the mean with standard deviation when normally distributed and the median with interquartile range (IQR) when nonnormally distributed. Comparisons between the GH group and the RTC group were performed using the independent samples t test for normally distributed continuous variables and the Mann‒Whitney U test for ordinal and nonnormally distributed continuous data. Proportions were compared using the Pearson χ^2^ test, and the exact z-pooled test when the expected count was less than 5 for any cell, as recommended [21]. Cases with missing values were not included in the analyses. Proportions are reported as a percentage of the total included cases, regardless of the missing status, except for reported CT findings. All CT variables were missing in seven patients, and proportions of all cases with nonmissing status were reported. Missing data are noted in all tables, except when the frequency of missing values was < 5%. The significance level was set to *α* = 0.05. All statistical analyses were performed using IBM SPSS Statistics version 25.

## Results

### Included patients

**Fig. 1:**
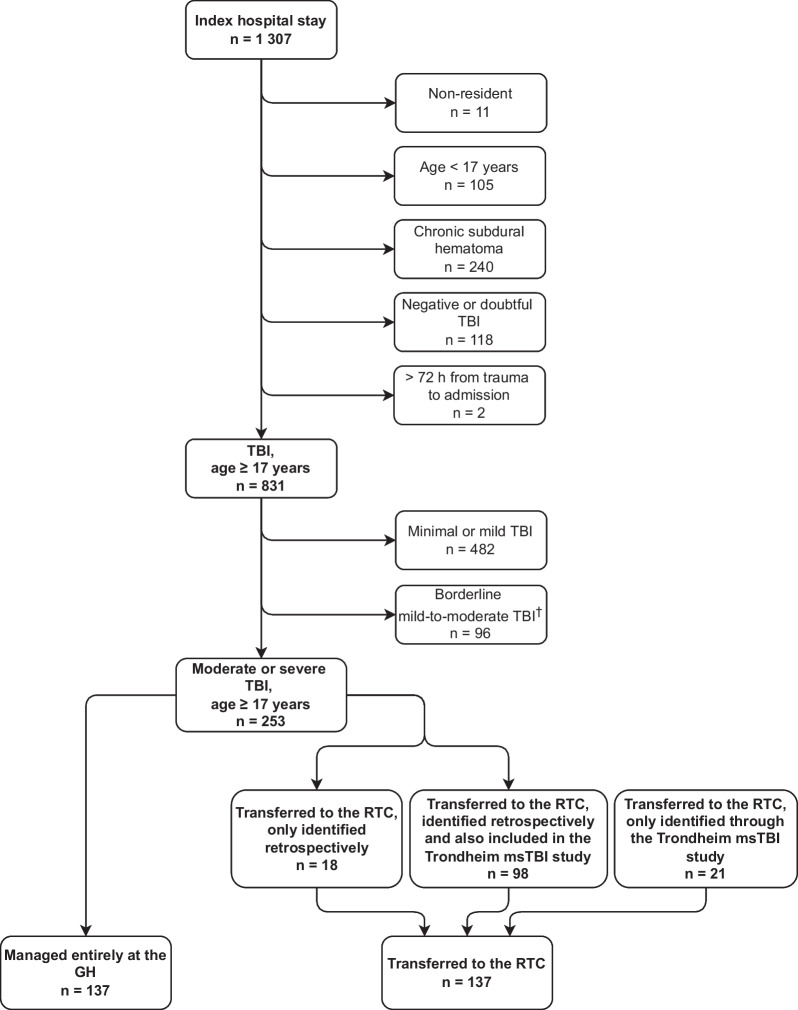
Flowchart of the inclusion process. Index hospital stay refers to any admission to a general hospital with ICD-10 code S06.1-S06.9, S09.7-S09.9 during the study period. *GH* General hospital, *msTBI* moderate and severe TBI*, RTC* Regional trauma centre, *TBI* Traumatic brain injury. ^**†**^Categorised as borderline mild-to-moderate due to the inability to accurately classify these patients’ TBI severity. For instance, intoxicated patients with an initially low GCS score who regained normal consciousness within 12 h, and severely demented patients with an already reduced GCS score preinjury

In total, 1307 index hospital stays at the seven GHs were reviewed. Of these, 253 patients met the inclusion criteria for moderate or severe TBI (including deterioration from mild TBI in the acute phase). In addition, 19 patients were included from the Trondheim msTBI study’s database (Fig. 1). These were not in the retrieved lists of ICD-10 codes, for instance, because very short stays at the GH had not been coded or because other ICD-10 codes had been used, for instance, I61 intracerebral haemorrhage. Hence, the total study population was 274 patients.

The overall transferral rate was 50%, leaving 137 in the GH group and 137 in the RTC group. There were no major differences between the seven GHs in transferral rate (Additional file 1: Table S1). The transferral rate was 58% for patients with severe TBI (n = 125) and 40% for patients with moderate TBI (n = 119). The patients transferred to the RTC had a median length of stay of 2.8 h (IQR 1.9–5.2 h) at the GH before transferral.

### Patient characteristics, fatality, and discharge destination in the GH and RTC groups

There were more patients with moderate TBI (52% vs. 35%, *p* = 0.005) in the GH group. The patients in the GH group were significantly older than the patients in the RTC group (median age: 78 years vs. 54 years, *p* < 0.001) and were more often injured in falls (Table 1). In addition, patients in the GH group more often had preinjury disability than did patients in the RTC group (50% vs. 39%, *p* = 0.037) and more often received antithrombotic medication (48% vs. 28%, *p* < 0.001). Platelet inhibitors were the most common antithrombotic medication for both groups (Additional file 1: Table S2). Anticoagulants were used by 14% of patients in the GH group and 9% in the RTC group (*p* = 0.106) (Table 1). In the GH group, fewer patients were admitted with TTA (35% vs. 57%, *p* < 0.001), received extracranial surgery (13% vs. 32%, *p* < 0.001), or were admitted to the ICU (80% vs. 93%, *p* < 0.001).

**Table 1:** Patients characteristics in the GH group and the RTC group (n = 274)

	GH group	RTC group^a^	*p*-value
	n = 137	n = 137	
Age in years, median [IQR]	78 [55, 87]	54 [33, 68]	**< 0.001**
Age in years, mean (SD)^b^	68 (23)	52 (21)	N/A
Patients ≥ 80 years old, n (%)	57 (42)	11 (8)	**< 0.001**
Male, n (%)	90 (66)	108 (79)	**0.015**
Preinjury functional disability, n (%) yes (%)	69 (50)	54 (39)	**0.037**
Missing	8 (6)	4 (3)	N/A
Antithrombotic medication, n (%)	66 (48)	38 (28)	**< 0.001**
Missing	11 (8)	1 (1)	N/A
Anticoagulant use, n (%)	19 (14)	12 (9)	0.106
Missing	13 (9)	1 (1)	N/A
Alcohol intoxication, n (%)	25 (18)	44 (32)	**0.009**
Cause of injury, n (%)			
Fall	111 (81)	75 (55)	**< 0.001**
Motor vehicle accident	17 (12)	41 (30)	**< 0.001**
Other cause	7 (5)	17 (12)	N/A
Admission GCS score, median [IQR]	10 [5, 12]	8 [5, 13]	0.827
Missing, n (%)	30 (22%)	4 (3%)	N/A
Admission GCS score of 13, n (%)	13 (9%)	17 (12%)	0.883
Severity of TBI at admission, n (%)			
Severe TBI	53 (39)	72 (53)	**0.021**
Moderate TBI	71 (52)	48 (35)	**0.005**
Mild TBI	13 (9)	17 (12)	0.439
Trauma team activation, n (%)	48 (35)	78 (57)	**< 0.001**
Extracranial injury, n (%)	58 (42)	68 (50)	0.224
Extracranial surgery, n (%)	18 (13)	44 (32)	**< 0.001**
ICU stay, n (%)	109 (80)	128 (93)	**< 0.001**
Neurosurgical procedure, n (%)	–	94 (69)	–

Number of missing cases not stated when frequency < 5%

*GCS* Glasgow coma scale, *GH* General hospital, *IQR* Interquartile range, *N/A* Not applicable, *RTC* Regional trauma centre, *SD* Standard deviation, *TBI* Traumatic brain injury

Bold font indicates statistical significance

^a^Two patients were transferred to a different RTC than St. Olavs Hospital due to extraordinary circumstances

^b^Statistical analyses were not conducted due to non-normality in the two groups

EDH, multiple contusions, and general oedema were more frequent in the RTC group (*p* < 0.001, *p* = 0.026 and *p* < 0.001, respectively Table 2). SDH and tSAH were frequent in both groups. In the RTC group, 94 patients (69%) had a neurosurgical procedure (Table 1).

**Table 2:** Main findings at head CT scans in the GH group and the RTC group

	GH group	RTC group	*p*-value
	n = 131^a^	n = 136^b^	
Normal first CT, n (%)	2 (1)	1 (1)	0.540
Epidural haematoma, n (%)	6 (4)	26 (19)	**< 0.001**
Subdural haematoma, n (%)	92 (67)	93 (68)	0.744
Subarachnoid haemorrhage, n (%)	92 (67)	90 (66)	0.477
Single contusion/haematoma, n (%)	33 (24)	18 (13)	**0.013**
Multiple contusions/haematomas, n (%)	45 (33)	65 (47)	**0.026**
Intraventricular haemorrhage, n (%)	32 (23)	36 (26)	0.702
General oedema, n (%)	4 (3)	48 (35)	**< 0.001**
Skull fracture without impression, n (%)	53 (39)	72 (53)	**0.041**
Skull fracture with impression, n (%)	5 (4)	10 (7)	0.210
Intracranial air, n (%)	18 (13)	39 (28)	**0.003**

*CT* Computed tomography, *GH* General hospital, *RTC* Regional trauma centre

Bold font indicates statistical significance

^a^There was missing data for 6 cases in the GH group, for all variables except epidural haematoma of which there were 7 missing cases

^b^There was missing data for 1 case in the RTC group, for all variables

More patients died in the acute phase in the GH group than in the RTC group (44% vs. 16%, *p* < 0.001) (Table 3) but less often from intracranial hypertension (70% vs. 95%, *p* = 0.015). The six-month case fatality rate (CFR) was higher in the GH group than in the RTC group (54% vs. 25%, *p* < 0.001).

**Table 3:** Fatality and discharge destinations in the GH group and the RTC group

	GH group	RTC group	*p*-value
	n = 137	n = 137	
Length of stay in days, median [IQR]	5 [2, 12]	6 [3, 12]	0.169
In-hospital fatality, n (%)	60 (44)	22 (16)	**< 0.001**
Cause of in-hospital fatality, n (%)	60 (44)	22 (16)	
Intracranial hypertension	42 (70)	21 (95)	**0.015**
Complications of TBI	17 (28)	1 (5)	**0.021**
Unrelated to the injury	1 (2)	0 (0)	N/A
Other trauma	0 (0)	0 (0)	N/A
Discharge destination, n (%)	77 (56)	115 (84)	
Nursing home	25 (32)	0 (0)	**< 0.001**
Home	22 (29)	2 (2)	**< 0.001**
Specialised rehabilitation	18 (23)	8 (7)	**0.001**
Other rehabilitative institution	6 (8)	0 (0)	N/A
Other hospital	6 (8)	105 (91)	**< 0.001**
6-month fatality, n (%)	74 (54)	34 (25)	**< 0.001**
Missing	0 (0)	8 (6)	N/A
12-month fatality, n (%)	79 (58)	38 (28)	**< 0.001**
Missing	0 (0)	8 (6)	N/A

*GH* General hospital, *IQR* Interquartile range, *N/A* Not applicable, *RTC* Regional trauma centre, *TBI* Traumatic brain injury

Bold font indicates statistical significance

Patients in the GH group were discharged to a variety of settings, including specialised rehabilitation (23%) and nursing homes (32%). In the RTC group, most (91%) were discharged to their respective GHs.

### Reasons for managing patients at the GH and relation to fatality

A consultant neurosurgeon or resident in neurosurgery on call at the RTC was consulted from the ED in 110 cases (80%) in the GH group. In addition, 12 cases (8%) were discussed later in the acute phase. All patients categorised as *unsalvageable* died during the acute phase (Table 4). Among patients for whom *neurosurgical intervention was considered nonbeneficial*, 17% presented with severe TBI at admission, and 90% died within 6 months. Among patients with a *presumed low risk of further deterioration*, four patients (8%) died within 6 months (age range: 71–94 years). These four patients died during the acute phase or were discharged to palliative care. One was later diagnosed with a severe concurrent somatic disease, while the remaining three patients were > 70 years old, none had preinjury disability, all had a mild TBI in the ED phase, and all were on antithrombotic medications.

**Table 4:** Reasons for managing patients entirely at the GH (n = 137), with corresponding severity of TBI and fatality

	Unsalvageable	Neurosurgical intervention considered non-beneficial	Presumed low risk of further deterioration	Another reason	Unknown reason
	n = 32 (23%)	n = 42 (31%)	n = 49 (36%)	n = 8 (6%)	n = 6 (4%)
Age, median [IQR]	79 [69, 85]	87 [82, 90]	58 [41, 71]	48 [37, 65]	43 [25, 78]
Patients ≥ 65 years old, n (%)	24 (75)	40 (95)	16 (33)	2 (25)	2 (33)
Patients ≥ 80 years old, n (%)	14 (44)	35 (83)	7 (14)	0 (0)	1 (17)
Severe TBI at admission, n (%)	31 (97)	7 (17)	8 (16)	4 (50)	3 (50)
Moderate TBI at admission, n (%)	1 (3)	31 (74)	32 (65)	4 (50)	3 (50)
Mild TBI at admission, n (%)	0 (0)	4 (10)	9 (18)	0 (0)	0 (0)
In-hospital fatality, n (%)	32 (100)	27 (64)	1^a^ (2)	0 (0)	0 (0)
6-month fatality, n (%)	32 (100)	38 (90)	4 (8)	0 (0)	0 (0)

*ED* Emergency department, *GH* General hospital, *IQR* Interquartile range, *TBI* Traumatic brain injury

^a^Two additional patients > 70 years of age were discharged to palliative care and died, thus fatal outcome was observed in the acute phase in 6% of patients in this category

### Elderly patients

The percentage of patients transferred to the RTC decreased with increasing age, from 64% of patients < 65 years old to 16% of patients ≥ 80 years old (Table 5). Patients ≥ 80 years old constituted 25% of the total study population: 42% of the GH group versus 8% of the RTC group (*p* < 0.001) (Table 1). In the GH group, 63% of the patients ≥ 80 years old had a preinjury functional disability, most commonly a neurologic condition.

**Table 5:** Characteristics of elderly patients in the GH group and the RTC group

	< 65 years old		≥ 65 years old		≥ 80 years old	
	GH group	RTC group	GH group	RTC group	GH group	RTC group
	n = 53	n = 95	n = 84	n = 42	n = 57	n = 11
Preinjury functional disability, n (%)	20 (38)	34 (36)	49 (58)	20 (48)	36 (63)	4 (36)
Missing	4 (8)	4 (4)	4 (5)	0 (0)	2 (4)	0 (0)
Antithrombotic medication, n (%)	5 (9)	8 (8)	61 (73)	30 (71)	41 (72)	9 (82)
Missing	6 (11)	1 (1)	5 (6)	0 (0)	3 (5)	0 (0)
Cause of injury, n (%)						
Fall	35 (66)*	43 (45)	76 (90)*	32 (76)	53 (93)*	7 (64)
Motor vehicle accident	11 (21)	32 (34)	6 (7)*	9 (21)	3 (5)**	4 (36)
Other cause	5 (9)	16 (17)	2 (2)	1 (2)	1 (2)	0 (0)
Severity of TBI at admission, n (%)						
Severe TBI	20 (38)*	53 (56)	33 (39)	19 (45)	20 (35)	7 (64)
Moderate TBI	32 (60)**	33 (35)	39 (46)	15 (36)	31 (54)***	0 (0)
Mild TBI	1 (2)	9 (9)	12 (14)	8 (19)	6 (11)	4 (36)
Trauma team activation, (%)	24 (45)*	59 (62)	24 (29)	19 (45)	12 (21)	4 (36)
Reason for managing patient at the GH, n (%)						
Unsalvageable	8 (15)	–	24 (29)	–	14 (25)	–
Neurosurgical intervention considered non-beneficial	2 (4)	–	40 (48)	–	35 (61)	–
Presumed low risk of further deterioration	33 (62)	–	16 (19)	–	7 (12)	–
Another reason	6 (11)	–	2 (2)	–	0 (0)	–
Unknown reason	4 (8)	–	2 (2)	–	1 (2)	–
Neurosurgical procedure, n (%)	–	70 (74)	–	24 (57)	–	6 (55)
In-hospital fatality, n (%)	8 (15)	12 (13)	52 (62)***	10 (24)	38 (67)	4 (36)
6-month fatality, n (%)	10 (19)	16 (17)	64 (76)***	18 (43)	48 (84)	8 (73)
Missing	0 (0)	7 (7)	0 (0)	1 (2)	0 (0)	0 (0)
12-month fatality, n (%)	11 (21)	18 (19)	68 (81)***	20 (48)	49 (86)	8 (73)
Missing	0 (0)	7 (7)	0 (0)	1 (2)	0 (0)	0 (0)

Number of missing cases not stated when frequency < 5%

*ED* Emergency department, *GCS* Glasgow coma scale, *GH* General hospital, *RTC* Regional trauma centre, *TBI* Traumatic brain injury

*p < 0.05, **p < 0.01, ***p < 0.001 in statistical analyses between the GH group and the RTC group for this age category

In the category *unsalvageable*, 75% of patients were ≥ 65 years old, and 44% of patients were ≥ 80 years old. In the category *neurosurgical intervention considered nonbeneficial*, 95% of patients were ≥ 65 years old, and 83% of patients were ≥ 80 years old (Table 4).

In patients ≥ 65 years old, the in-hospital CFR was higher in the GH group than in the RTC group (62% vs. 24%, *p* < 0.001), as was the 6-month CFR (76% vs. 43%, *p* < 0.001) (Table 5). In patients ≥ 80 years old, the in-hospital CFR seemed to be higher in the GH group than in the RTC group, although not significantly different (67% and 36%, *p* = 0.062), whereas the CFR at 6 months was similar (84% and 73%, *p* = 0.420).

## Discussion

This is the first Scandinavian study describing adult patients with moderate and severe TBIs admitted to GHs, focusing on patients never transferred to RTCs. Of all patients initially admitted to a GH, 60% of patients with moderate and 42% of patients with severe TBI were managed entirely at the GH. Most (94%) of the patients *presumed to have a low risk of further deterioration* remained stable and survived the acute phase. In addition to a large proportion of patients considered *unsalvageable* (23%), we found that the status *neurosurgical intervention considered nonbeneficial,* especially among elderly individuals with preinjury disability, was a common cause of not transferring the patient. More patients died in the acute phase in the GH group than in the RTC group, potentially reflecting the higher number of elderly patients in the GH group. Overall, half of the patients were managed entirely at the GH. The decision regarding the appropriate level of care for the patient was mostly made in cooperation with a neurosurgeon. Most patients with EDH, multiple contusions or general oedema were transferred. Most of the transferred patients received neurosurgical procedures, indicating that patients evaluated as likely to benefit from neurosurgery were transferred.

We identified two main categories of patients managed entirely at the GHs: patients considered safe to manage at the GH level and elderly patients considered either unsalvageable or unlikely to benefit from neurosurgical intervention.

First, few patients with a *presumed low risk of further deterioration* died during the acute phase, including patients with a GCS score ≤ 8 at admission, indicating that communication between the managing physician at the GH and the on-call neurosurgeon, with shared access to the CT scan, may provide an accurate estimate of the patient’s prognosis; this finding is in line with other studies [22, 23]. The four patients in this category who deteriorated and died or entered a palliative setting within six months after the injury were all elderly and receiving antithrombotic medication or were later diagnosed with a severe concurrent disease. It remains uncertain whether the four deaths could have been prevented by transferral to the RTC. This study extends the current knowledge regarding patients with moderate TBI [24], as we have demonstrated that 60% of the patients admitted to GHs were managed entirely at the GH. Several studies have investigated the opportunities for the management of patients with TBI without transfer to a neurosurgical department, but most of these studies have only included patients with mild TBI or have health care systems not readily comparable to the Scandinavian health care model [25–27]. On both the national and international levels, there is a lack of guidelines offering nuanced recommendations for the management of moderate and severe TBI at the GH level.

The second group included elderly patients considered either unsalvageable or unlikely to benefit from neurosurgical intervention. Patients managed entirely at the GH were older than patients transferred to the RTC, and only 16% of patients ≥ 80 years old were transferred to the RTC. Even among patients with severe TBI, almost half were never transferred to the RTC, despite a high degree of conferring with the RTC and current guidelines recommending transfer [4, 28]. The most common rationale for not transferring patients with severe TBI was that the patients were considered unsalvageable, and most (75%) of these were ≥ 65 years old. It is likely that younger patients, even those with no hope of survival, were admitted directly to or rapidly transferred to the RTC, for instance, when involved in road traffic accidents [29]. Nevertheless, even when excluding patients categorised as *unsalvageable*, one-fourth of patients with severe TBI were managed entirely at the GH, many of whom were elderly patients where *neurosurgical intervention was considered nonbeneficial*. Elderly patients in the GH group had a high rate of preinjury disability, which increased with increasing age. Age and comorbidity have been associated with less aggressive management [7, 30, 31] and lower rates of transferral [32–34] in previous studies, consistent with our findings.

The choice of management level for elderly patients has been insufficiently studied since many studies exclude elderly patients with preinjury disability or significant comorbidity [35]. One study did not find an association between transfer to a level I or II trauma centre and long-term functional outcomes in older patients with all-severity TBI and concluded that routine transfer is not warranted [36]. Considering the increasingly older population in high-income countries and a demonstrated increase in TBIs in elderly individuals, this is an important topic that needs further investigation [7].

CFRs were high in the elderly patients in the GH group for both patients≥ 65 years and ≥ 80 years old but also in patients ≥ 80 years old in the RTC group. Studies regarding the benefits of neurosurgical intervention in the elderly are conflicting [37–42]. Moreover, these studies were conducted at neurosurgical referral centres and were likely biased towards cases considered suitable for surgical intervention. The in-hospital CFR was higher for patients ≥ 80 years old in the GH group versus the RTC group; however, the 6-month CFR was very high in both groups. This increase in fatality rate during the first few months demonstrates that studies on elderly patients must also consider post-discharge status when investigating outcomes.

Whether the high CFR among elderly patients indicates a nihilistic approach or well-considered treatment-limiting decisions was not investigated in this study. The former possibility has been explored in several previous studies, which found that less aggressive management in elderly patients might increase mortality rates [31, 43]. In the latter study, increasing age was associated with reduced management intensity, irrespective of head injury severity, and an association between low management intensity and increased risk of 30-day mortality was found. However, in some cases, palliative care would be in the best interest of the patient [44], and our study shows that treatment-limiting decisions are common for elderly patients with severe TBI in our region, consistent with another Norwegian study [45]. Interestingly, a German and Austrian study found that even if neurosurgeons were willing to perform an emergency operation on an elderly patient with a life-threatening TBI [46], the elderly patients themselves might not wish for a life-prolonging intervention if it would involve severe disability [47].

As in geriatric medicine, the focus on frailty in the field of TBI has increased. Frailty reflects the individual’s physiological vulnerability beyond that of chronological age, and a TBI-specific frailty index was recently developed and validated [48]. Management guidelines for TBI could probably be improved by incorporating the assessment of frailty [49].

### Limitations

Although the population-based design, covering all the GHs without neurosurgical services in the region over a ten-year period, was a strength of this study, the retrospective nature of this study entails some limitations of the data material. First, the long inclusion period and the time passed since the last included patients, imply that the study results might not generalize to the current situation at general hospitals. Second, ICD-10 codes to be reviewed were limited as described in the methods section, thereby excluding cases coded with S06.0 (concussion). We might therefore have missed a few patients with a normal head CT scan who clinically suffered a moderate or severe TBI due to isolated TAI. We might also have missed cases due to incorrect coding [12]. Third, the GCS score was in some cases not documented or could not be validly obtained from the medical records, for instance, due to concomitant alcohol intoxication, extracranial severe injuries affecting respiratory or circulatory function, dementia, or other neurologic comorbidities. Moreover, reasons for managing patients entirely at the GH were not always accurately described and can be especially difficult to derive retrospectively, which was also the case for the cause of death; the results must therefore be interpreted with caution.

An additional limitation is that the present study used the HISS classification of moderate TBI (GCS scores of 9–13), instead of the range of 9–12 used in many current studies. Last, patients residing in the area where the RTC also serves as the GH were not included in this study. Consequently, the present study did not include all cases of moderate and severe TBI in central Norway; instead, it attempted to investigate the situation from the perspective of GHs without neurosurgical services.

## Conclusions

GHs manage many patients with moderate or severe TBI, and there is a high degree of communication between the GHs and the RTCs about these patients. The patients managed entirely at the GHs differed from the patients transferred to the RTC by being older and having more preinjury disabilities. Hence, studies conducted at hospitals with a neurosurgical department will not be representative of all patients with moderate or severe TBI. Two main groups of patients were identified in the GH group: patients considered to have a low risk of further deterioration—a mostly accurate prediction, and elderly patients dying upon arrival or presenting with severe comorbidities considered not to benefit from neurosurgical intervention due to assumed poor prognosis. Notably, few patients ≥ 80 years old were transferred to the RTC, and the 6-month CFR in this age group was high regardless of transferral. Transferral to the RTC may therefore not be in the best interest of older patients with high premorbid frailty. Future guidelines should describe the management of moderate and severe TBI in the elderly population in greater detail, especially regarding the treatment level and benefit of transferral.

## Supplementary Information

**Additional file 1: Table S1** Patients with moderate or severe TBI in the GHs of central Norway 01.10.2004–01.10.2014. **Table S2** Preinjury functional disability and antithrombotic medication usage in the GH group versus the RTC group.

## Data Availability

The datasets used and/or analysed during the current study are available from the corresponding author on reasonable request.

## Abbreviations

BTF: Brain Trauma Foundation
CFR: Case fatality rate
CT: Computed tomography
ED: Emergency department
EDH: Epidural haematoma
GCS: Glasgow Coma Scale
GH: General hospital
ICD-10: International Classification of Diseases, 10th revision
ICP: Intracranial pressure
ICU: Intensive care unit
msTBI: Moderate and severe TBI
RTC: Regional trauma centre
tSAH: Traumatic subarachnoid haemorrhage
SDH: Subdural haematoma
SNC: Scandinavian Neurotrauma Committee
TAI: Traumatic axonal injury
TBI: Traumatic brain injury
TTA: Trauma team activation
