# Supplementary material for: Moderate and severe traumatic brain injury in general hospitals: a ten-year population-based retrospective cohort study in central Norway
Source: Scand J Trauma Resusc Emerg Med. 2022 Dec 9;30:68. doi: 10.1186/s13049-022-01050-0 (PMC9733333; doi:10.1186/s13049-022-01050-0)
Supplement: Supplementary file 1 — Additional file 1: Table S1 Patients with moderate or severe TBI in the GHs of central Norway 01.10.2004–01.10.2014. Table S2 Preinjury functional disability and antithrombotic medication usage in the GH group versus the RTC group. [file 13049_2022_1050_MOESM1_ESM.docx]

Moderate and severe traumatic brain injury in general hospitals – a ten-year population-based retrospective cohort study in central Norway

Additional file 1: Supplementary Tables S1 and S2

**Supplementary Table S1** Patients with moderate or severe TBI in the GHs of central Norway 01.10.2004-01.10.2014.

|  | **Ålesund  n = 110** | **Molde  n = 40** | **Kristian-sund**^a^ **n = 27** | **Volda**^b^  **n = 24** | **Levanger  n = 38** | **Namsos  n = 18** | **Orkdal**^c^  **n = 17** | **Total  N = 274** |
| --- | --- | --- | --- | --- | --- | --- | --- | --- |
| **Approximate catchment**  **(in 1 000 citizens)** | 100 | 60 | 50 | 50 | 100 | 40 | 70^d^ |  |
| **Age, median [IQR]** | 62 [42, 79] | 56 [31, 77] | 51 [32, 64] | 74 [35, 84] | 77 [58, 86] | 69 [44, 84] | 77 [44, 83] | 63 [42, 80] |
| **Managed at the GH  level, n (%)** | 55 (50) | 20 (50) | 10 (37) | 15 (63) | 20 (53) | 10 (56) | 7 (41) | 137 (50) |
| **Severe TBI, n (%)** | 62 (56) | 15 (38) | 12 (44) | 7 (29) | 15 (39) | 6 (33) | 8 (47) | 125 (46) |
| **In-hospital fatality, n (%)** | 33 (30) | 10 (25) | 7 (26) | 8 (33) | 12 (32) | 6 (33) | 6 (35) | 82 (30) |

**Abbreviations: *GH* General hospital, *IQR* Interquartile range, *TBI* Traumatic brain injury**

^a-c^All GHs were acute care hospitals for nearly all of the study period; Kristiansund Hospital and Volda Hospital until May 2014 and Orkdal Hospital until August 2011.

^d^Orkdal hospital receives fewer trauma patients than its catchment population indicates due to many trauma patients being admitted directly to the RTC, St. Olavs Hospital, after special arrangement.

**Supplementary Table S2** Preinjury functional disability and antithrombotic medication usage in the GH group versus the RTC group.

|  | | **GH group n = 137** | **RTC group n = 137** | ***p*-value** |
| --- | --- | --- | --- | --- |
| **Preinjury functional disability, n (%)** | | 69 (50) | 54 (39) | **0.037** |
|  | Missing | 8 (6) | 4 (3) |  |
| **Predominant disease category in disability, n (%)** | | 69 (50) | 54 (39) |  |
|  | Neurologic condition  n (%) | 30 (43) | 12 (22) |  |
|  | Alcohol abuse | 14 (20) | 20 (37) |  |
|  | Cardiopulmonary disease | 8 (12) | 3 (6) |  |
|  | Cancer | 3 (4) | 0 (0) |  |
|  | Psychiatric disorder | 2 (3) | 11 (20) |  |
|  | Substance abuse | 2 (3) | 1(2) |  |
|  | Developmental disorder n (%) | 0 (0) | 1 (2) |  |
|  | Other | 1 (1) | 2 (4) |  |
|  | Several | 9 (13) | 4 (7) |  |
| **Antithrombotic medication, n (%)** | | 66 (48) | 38 (28) | **< 0.001** |
|  | Missing | 11 (8) | 1 (1) |  |
| **Type of antithrombotic medication, n (%)** | | 66 (48) | 38 (28) |  |
|  | Platelet inhibitors | 49 (74) | 26 (68) |  |
|  | Anticoagulants | 19 (29) | 12 (32) |  |
|  | Both | 2 (3) | 0 (0) |  |

**Abbreviations: *GH* General hospital, *RTC* Regional trauma centre**
